# Telomere Dysfunction Is Associated with Altered DNA Organization in Trichoplein/Tchp/Mitostatin (TpMs) Depleted Cells

**DOI:** 10.3390/biomedicines10071602

**Published:** 2022-07-05

**Authors:** Angela Lauriola, Pierpaola Davalli, Gaetano Marverti, Andrea Caporali, Sabine Mai, Domenico D’Arca

**Affiliations:** 1 Department of Biotechnology, University of Verona, 37134 Verona, Italy; angela.lauriola@univr.it; 2 Department of Biomedical, Metabolic and Neural Sciences, University of Modena and Reggio Emilia, Via G. Campi 287, 41125 Modena, Italy; pierpaola.davalli@unimore.it (P.D.); gaetano.marverti@unimore.it (G.M.); 3 The Queen’s Medical Research Institute, BHF Centre for Cardiovascular Science, University of Edinburgh, Edinburgh EH10 4AH, UK; acaporal@exseed.ed.ac.uk; 4 CancerCare Manitoba Research Institute, University of Manitoba, CancerCare Manitoba, Winnipeg, MB R3E 0V9, Canada

**Keywords:** mitostatin, cancer, telomeres, structured illumination microscopy, nuclear architecture, quantitative microscopy

## Abstract

Recently, we highlighted a novel role for the protein Trichoplein/TCHP/Mitostatin (TpMs), both as mitotic checkpoint regulator and guardian of chromosomal stability. TpMs-depleted cells show numerical and structural chromosome alterations that lead to genomic instability. This condition is a major driving force in malignant transformation as it allows for the cells acquiring new functional capabilities to proliferate and disseminate. Here, the effect of TpMs depletion was investigated in different TpMs-depleted cell lines by means of 3D imaging and 3D Structured illumination Microscopy. We show that TpMs depletion causes alterations in the 3D architecture of telomeres in colon cancer HCT116 cells. These findings are consistent with chromosome alterations that lead to genomic instability. Furthermore, TpMs depletion changes the spatial arrangement of chromosomes and other nuclear components. Modified nuclear architecture and organization potentially induce variations that precede the onset of genomic instability and are considered as markers of malignant transformation. Our present observations connect the tumor suppression ability of TpMs with its novel functions in maintaining the proper chromosomal segregation as well as the proper telomere and nuclear architecture. Further investigations will investigate the connection between alterations in telomeres and nuclear architecture with the progression of human tumors with the aim of developing personalized therapeutic interventions.

## 1. Introduction

TpMs (Trichoplein/TCHP/Mitostatin) was identified in well-differentiated epithelial cells as a protein associated with many types of keratins, being involved in their organization, and was named as “trichoplein keratin filament binding protein” [1]. TpMs protein is localized in the cell cytoplasm, in the cytoskeleton and desmosomes of polarized cells [1], and mitochondria [2]. Keratin filaments bind desmosomes at the plasma membrane and extend into the cytoplasm to produce a scaffold for cytoskeletal elements such as the centrosome. TpMs protein is situated near the distal ends of the mother centriole and binds to the centrosomal proteins Odf2 and ninein, thereby controlling microtubule anchoring to the centrosome [3,4]. Nishimura et al. emphasize how TpMs regulates the cell cycle through primary cilium formation [4]. TpMs activates the mitotic kinase Aurora A through a direct molecular interaction during centrioles activation at the G1-phase, thus negatively regulating primary cilia assembly in this phase [5]. TpMs is involved in the morphological and ultrastructural organization of mitochondria [2], in the endoplasmic reticulum-mitochondria juxtaposition [6] and in the mitophagy evoked by the decorin protein [7]. We found that TpMs links autophagy with endothelial cell functions, as it regulates cell functions via an autophagy-mediated mechanism [8]. TpMs protein is evolutionarily conserved among several species and is detectable at different levels in various human tissues. The gene encoding TpMs localizes to the chromosome 12q24.1; it was identified as Ts12q and a putative tumor suppressor at 12q [2]. Our previous investigations demonstrated that TpMs silencing causes abnormal activity of the spindle assembly checkpoint (SAC) in several cell lines [9]. SAC represents a cell cycle checkpoint during mitosis and meiosis, which allows the proper segregation of chromosomes by preventing the separation of the duplicated chromosomes during the anaphase until each chromosome is properly attached to the spindle. TpMs-depleted cancer cells show numerical/structural chromosome instability as well as increased numbers of chromosome bridges and lagging chromosomes. These aberrations are typical hallmarks of chromosome mis-segregation, premature sister–chromatid separation and chromosomal end-to-end fusions, which are all distinctive features of chromosomal instability (CIN). CIN is characterized by the gain or loss of chromosomes, as well as by the rearrangement of the genetic material during the cell division, finally generating ongoing and dynamic genomic instability [9]. CIN is considered a major driving force in human malignant transformation, leading cancer cells of different tissues in different organs to acquire functional capabilities that allow them to survive, proliferate and disseminate [10,11,12].

Here, we investigated whether TpMs silencing causes dysfunction and alterations in telomeres, eventually resulting in CIN. Capping the chromosomes, telomeres are responsible for chromosomal integrity to prevent genomic instability. Each cell division causes gradual shortening of telomeres, leading normal cells to senescence or apoptosis. Telomere shortening is a feature of molecular aging and is associated with the premature appearance of diseases associated with aging [13,14,15]. The length of telomeres is maintained by telomerase in Protozoa as well as in Metazoans. In humans, the enzyme expression is highest in germline and progenitor compartments of cells. Normal cells maintain an optimum level of telomerase to support tissue homeostasis. During cancer development, alterations in telomeres are known to occur that represent one of cancer cells hallmarks [16,17,18]. More than 90% of nearly all human cancers show upregulation/reactivation of telomerase that supports the enhanced cell proliferation associated with carcinogenesis. The increased telomerase activity may confer cell immortalization, thereby facilitating tumor invasion and metastasis. A small group of cancers (~10%) exhibit a telomerase-independent mechanism of telomere maintenance, termed alternative lengthening of telomeres (ALT), which results in long and heterogeneous telomeres in the absence of telomerase expression. Both increased telomerase activity and ALT are telomere maintenance pathways that prevent cancer cells from undergoing cell death or senescence, although they may have short telomeres [19,20,21,22,23]. Occasionally, both telomerase and ALT co-activation have been reported [24,25]. Genetic and epigenetic alterations of the telomere maintenance machinery generate genome instability in carcinogenesis [21,22,23,26,27,28]. The telomere structure is dynamic and may be important for both transcriptional processes and for stabilizing the chromosome positions in the nucleus. A non-random and dynamic, cell-cycle-dependent and tissue-dependent three-dimensional (3D) organization of telomeres occurs in the nuclei to assure genomic stability [29]. Cancer is characterized by multiple alterations that affect the modulation of gene expression and the stability of the genome [30]. Interconnected changes occur in cell nuclei that alter their 3D organization during tumor initiation and progression. The 3D analysis of the genome organization in the interphase nucleus makes it possible to ascertain the level of genomic stability in both normal and cancer cells [29]. During carcinogenesis, the 3D architecture of telomeres is remodeled, as defined by diminished or increased telomeres number and length, increased numbers of telomere aggregates, modified telomere distribution within the nucleus, and altered 3D telomere positions [29,31].

Here, we show that TpMs depletion causes alterations in the 3D architecture of the telomeres in colon cancer HCT116 cells, but not in normal prostatic PNT1A cells. Moreover, considering that modified nuclear architecture is a marker of malignant transformation [32], we investigated whether TpMs depletion changes the genome organization of the cells through the alteration of the nuclear architecture organization, as defined by the spatial arrangement of chromosomes and other nuclear components. Altogether, we demonstrate that the telomere alterations generated in TpMs-depleted HCT116 cells are consistent with chromosome aberrations that generate chromosomal instability [9]. Our observations represent a connection between the tumor suppression ability of TpMs with its function in maintaining chromosomal segregation.

## 2. Materials and Methods

### 2.1. Cell Culture and Reagents

HCT116 and PNT1A cell lines were grown in DMEM-high Glucose medium and RPMI 1640, respectively, both supplemented with 10% fetal bovine serum (FBS). The cells were maintained in an incubator in a humidified atmosphere containing 5% CO_2_ at 37 °C. The cell lines were obtained from the ATCC and European collection of cell cultures, ECACC via Sigma. All the studies were performed in Mycoplasma negative cells, as routinely determined with the MycoAlert (Mycoplasma detection kit—Lonza, Walkersville, MD, USA) using the reagent DAPI (4′,6-diamidino-2-phenylindole) and a Cy3-labelled PNA probe (DAKO, Glostrup, Denmark).

### 2.2. Lentivirus Production and Infection

Lentiviral particles were generated by transient transfection into 293T cells. The shRNA-TpMs vector (#1 TRCN0000127662, #2 TRCN0000130868, Sigma Aldrich, St Louis, MO, USA) or the control vector (pLKO.1-puro) was co-transfected with the pCMV–VSV-G construct and the packaging construct pCMV-ΔR8.2Δvpr using calcium phosphate-mediated transfection. The shRNA vectors targeting the TpMs virus were collected at 48 hr post-transfection. The harvested supernatants were filtered through a 0.45 μm syringe filter. The concentration of lentiviral particles was obtained using Polyethylene Glycol 8000 (PEG8000). The HCT116 and PNT1A cells were infected, and the lentiviral integration was selected with 1 μg/mL puromycin. An average of 70% of the cells from each infection survived selection.

### 2.3. Three-Dimensional Quantitative Fluorescent In Situ Hybridization (3D Q-FISH)

The HCT116 and PNT1A cell lines were seeded on sterile pre-labelled microscope slides to perform the quantitative fluorescence in situ hybridization (Q-FISH) in interphase nuclei. The cells were first fixed with 3.7% formaldehyde/1 × PBS for 20 min and then washed three times with 1 × PBS. The cells were permeabilized by incubation in 0.5% Triton X-100 for 10 min and a freeze-thaw treatment was applied to preserve the 3D structure of the nucleus: This included soaking the slides in 20% glycerol, then dipping them into liquid nitrogen for a few seconds and allowing them to thaw; this procedure was repeated three times. Then, the slides were washed three times in 1 × PBS for 5 min before incubation in a 0.1 M HCl solution for 10 min. The hybridization was performed with 5 µL of telomeric peptide-nucleic acid (PNA) probe conjugated to a Cy3 fluorophore (DAKO, Glostrup, Denmark). The telomeric probe was applied to the area containing cells, which was covered with a coverslip and sealed with rubber cement. Using a HYBrite Denaturation and Hybridization System (Vysis; Abbott Diagnostics, Des Plains, IL, USA), denaturation was performed at 80 °C for 3 min, followed by probe hybridization at 30 °C for 2 h. The slides were then washed in 70% formamide (Fluka; Sigma-Aldrich, St Louis, MO, USA)/10 mM Tris (pH 7.4) twice for 15 min each, followed by a wash of 5 min in 0.1× saline sodium citrate buffer (SSC) at 55 °C, and two 5 min washes in 2xSSC/0.05% Tween. DAPI staining was performed with 50 µL of 0.1 µg/mL DAPI (4′, 6-diamidino-2-phenylindole) at room temperature for 3 min in the dark. Excess DAPI was rinsed with water and the slides were mounted with 22 × 22 mm coverslips (Zeiss, Toronto, ON, Canada) using a drop of Vectashield mounting medium (Vector Laboratories, Burlington, Toronto, ON, Canada).

### 2.4. Widefield 3D Imaging and Analysis

After the telomere Q-FISH was performed, the interphases were imaged by using a Zeiss AxioImager Z1 microscope with a cooled AxioCam HRB&W camera, DAPI, and Cy3 filters in combination with a Planapo 63.3/1.4 oil objective lens. The images were acquired using Axiovision 4.6 software (Zeiss) in multichannel mode followed by constraint iterative deconvolution as specified below. For every fluorochrome, the 3D image consisted of a stack of 80 images with a sampling distance of 200 nm along the z-axis and 107 nm in the x- and y-axis directions. In each sample, 35 nuclei were selected for analysis for each of the three replicates in each experiment. Images of the selected nuclei and videos were processed using an iterative deconvolution algorithm in the Axiovision software (Carl Zeiss, Jena, Germany), which removes out of focus light. The deconvolved images were exported as TIF files for analysis with the TeloviewTM v1.03 software program (Telo Genomics Corp., Toronto, ON, Canada). TeloviewTM was used to assess telomere numbers, signal intensity distribution, spatial distribution, and the presence of telomere aggregates.

### 2.5. SIM Images Acquisition and Analysis

For the Three-Dimensional Structured Illumination Microscopy (3D-SIM), we used the same slide preparation as used for the Q-FISH. The slides were washed in 1 × PBS for 5 min and 50 μL of 0,1 µg/mL DAPI (4′,6-diamidino-2-phenylindole) was added and the slides were incubated for 5 min. The excess DAPI was rinsed with water and drained. The coverslips were mounted on the slides with one drop of Vectashield mounting medium (Vector Laboratories, Burlington, Toronto, ON, Canada) and sealed with clear nail polish. The slides were stored at 4 °C until imaging.

### 2.6. 3D-SIM Imaging and Analysis

The cells were imaged with a Zeiss Elyra PS1 SIM equipped with a Plan-Apochromat 63/1.4 Oil DIC M27 and captured with an Andor EM-CCD iXon 885 camera with a 1.6× tube lens (all from Carl Zeiss, Toronto, ON, Canada). The DAPI channel was obtained with 405 nm laser excitation, 23 µm diffraction grating and filter cube SR Cube 07. The lateral pixel size, Dx and Dy, was 79 nm in the recorded images and 40 nm in the reconstructed image. Cell nuclei were chosen randomly and imaged for each of three replicates of the experiment. At least 30 cells for each slide were analyzed (three experiments were performed). The image processing was performed in MATLAB (MathWorks, Natick, MA, USA). A field of view was selected, and the z-stack boundaries were defined manually. The 3D-SIM and widefield images were reconstructed with ZEN 2012 black edition (Carl Zeiss, Jena, Germany) with the standard settings. The granulometry of the DNA structure and the structure of DNA-poor space was measured with a morphological sieve applied to the error-function clipped images [33]. The coefficient of variation and the skewness of the intensity histogram over the detected region were also calculated.

### 2.7. Western Blot

Cells were harvested in PBS using a cell scraper and washed once with PBS by centrifugation. The cell pellets were lysed for 30 min on ice in RIPA buffer (Cell signaling #9806) containing protease and phosphatase inhibitors. The cell proteins were quantified using the Bradford assay (Sigma-Aldrich B6913). Equal amounts of proteins were loaded onto SDS-Polyacrylamide gels and blotted on PVDF membranes (Amersham). The membranes were blocked in 5% non-fat milk in 0.1% TBST. The primary antibody incubation was performed in blocking solution at 4 °C, overnight. We used the following primary antibodies: anti-TpMs (Santa Cruz sc515025) and anti-β-Actin (Millipore MAB1501). Horseradish peroxidase (HRP)-conjugated anti-mouse (Sigma-Aldrich A5906) secondary IgG antibodies were used. The signal of the immunoblotted proteins was visualized using the ECL Plus Western Blotting Detection System (GE Healthcare Biosciences, Freiburg, Germany). The pixel intensity/quantification was performed using ImageJ.

### 2.8. Statistical Analysis

For statistical analysis, Chi-square tests were used to compare the percentage of interphase telomere signals. For the 3D-SIM imaging data, the distributions were compared using two-sided, two-sample Kolmogorov–Smirnov (KS) tests to determine the significance of difference. *p*-values < 0.05 were considered statistically significant.

## 3. Results

### 3.1. Telomere Alteration Increases in Tpms-Depleted Colon Cancer HCT116 Cells but Not in Tpms-Depleted Normal Prostatic Epidermal pnt1a Cell Line

**Figure 1:**
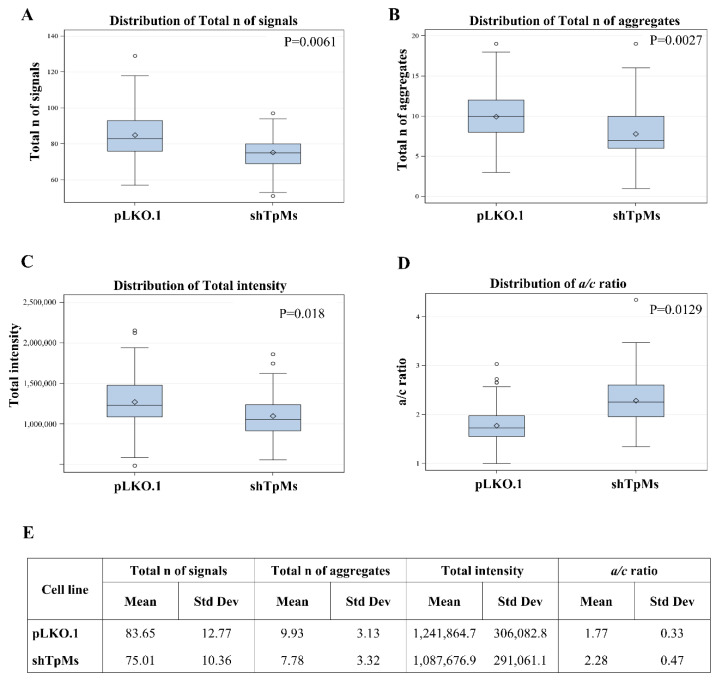
Loss of TpMs induces telomere dysfunction. Analysis of 3D nuclear telomere using TeloView in TpMs-depleted HCT116 cells shows a decrease in distribution of the total number of signals (*p* = 0.0061) (**A**); distribution of total numbers of aggregates (*p* = 0.0027) (**B**); distribution of total Intensity (*p* = 0.018) (**C**), and increase in distribution of a/c ratio (*p* = 0.0129) (**D**) compared with control cells (pLKO.1). The a/c ratio is defined as the nuclear space occupied by telomeres, represented by three axes of length a, b and c. The ratio between the a and c axes, the a/c ratio, reflects the distribution of telomeres, which changes at different stages of the cell cycle. (**E**) Mean = average of three different experiments. Std Dev = standard deviation.

**Figure 2:**
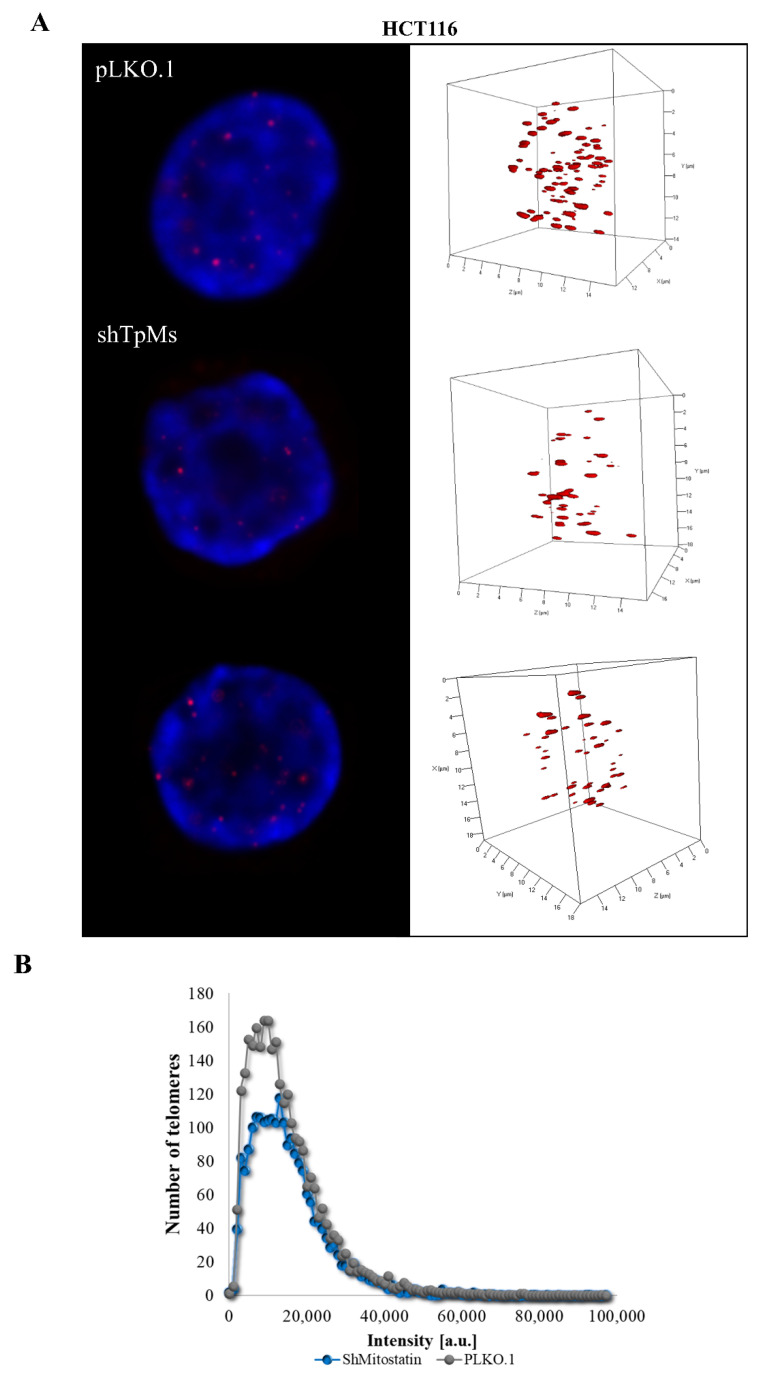
The number of telomere signals and aggregates decreases in TpMs-depleted HCT116 cells. (**A**) Representative nuclei, counterstained with DAPI (4′,6-diamidino-2-phenylindole) (blue), where Cy-3 labelled telomeres appear as red dots. TpMs-depleted HCT116 cells show a decreased number of telomere signals and aggregates (middle and bottom rows) compared with control cells, pLKO.1 (top row). (**B**) A telomere intensity histogram showing distribution of signal intensities in HCT116 cells depleted of TpMs with respect to the control. [a.u.] = arbitrary units. Abscissa = intensity [a.u]. ordinate = number of telomere signals.

Telomere alteration causes a spectrum of mitotic defects with anaphase bridges, triggering both numerical and structural chromosome changes that lead to chromosomal instability [18]. To investigate whether TpMs depletion induces telomere dysfunction, we analyzed the structure of telomeres in the colon cancer cell line (HCT116) and in the prostatic epithelial cell line PNT1A. The latter are normal cells that have been immortalized by the SV40 genome with a defective replication origin and are able to maintain a constant telomere length. Immortal cells are known to be not susceptible to telomere dysfunctions [34,35]. The telomere length was measured using the Q-FISH technique with a telomere-specific probe. We performed telomere analysis of 95 interphase nuclei using TeloView software. The TpMs-depleted cells (shTpMs HCT116, and shTpMs PNT1A) were generated by a lentiviral transduction of short hairpins targeting the TpMs coding sequence, as described in Section 2, and in previously published studies [8,9]. The reduced TpMs protein levels (sh TpMs) respect to control (pLKO.1) are shown in Figures S1 and S2 in Supplementary Materials. In the TpMs-depleted HCT116 cells we found that the number of telomere signals was lower than in the control cells (pLKO.1) (*p* = 0.0061) (Figure 1A,E and Figure 2 and Video S1). We also evaluated the numbers of telomere aggregates (Tas). Tas are fused telomeres or telomeres in close vicinity that cannot be further resolved into separate signals (telomeres) at the optical resolution limit of 200 nm, and represent telomere clusters [36].

Interestingly, we observed that the number of Tas decreased in the TpMs-depleted cells (*p* = 0.0027) (Figure 1B,E). We assessed telomere intensity, which is proportional to telomere length [37] and found a decrease in total telomere intensity and length (*p* = 0.018) (Figure 1C,E). Next, we defined the spatial distribution of telomeres in the nucleus, which is reflected by the a/c ratio. An increase in the a/c ratio in the TpMs-depleted HCT116 cells was demonstrated (*p* = 0.0129) (Figure 1D,E). These findings show that telomere dysfunction is increased in the absence of TpMs in the HCT116 cell line. The decreased telomere intensity is consistent with ongoing proliferation processes (mitotic slippage) observed in our recent publication [9]. In addition, the presence of telomere aggregates is indicative of ongoing genomic instability.

**Figure 3:**
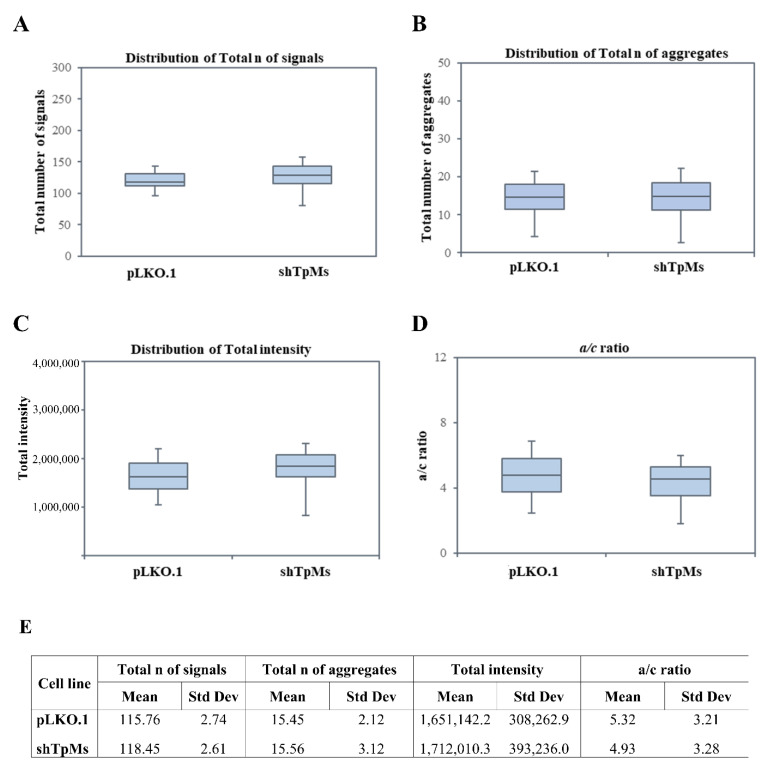
Loss of TpMs in PNT1A cells does not induce telomere dysfunction. Analysis of 3D nuclear telomere using TeloView in TpMs-depleted cells does not show significant differences in distribution of total number of signals (**A**); distribution of total number of aggregates (**B**); distribution of total intensity (**C**); and distribution of a/c ratio (*p* = 0.0129) (**D**) compared with control cells (pLKO.1). (**E**) Mean = average of three different experiments; Std Dev = standard deviation.

**Figure 4:**
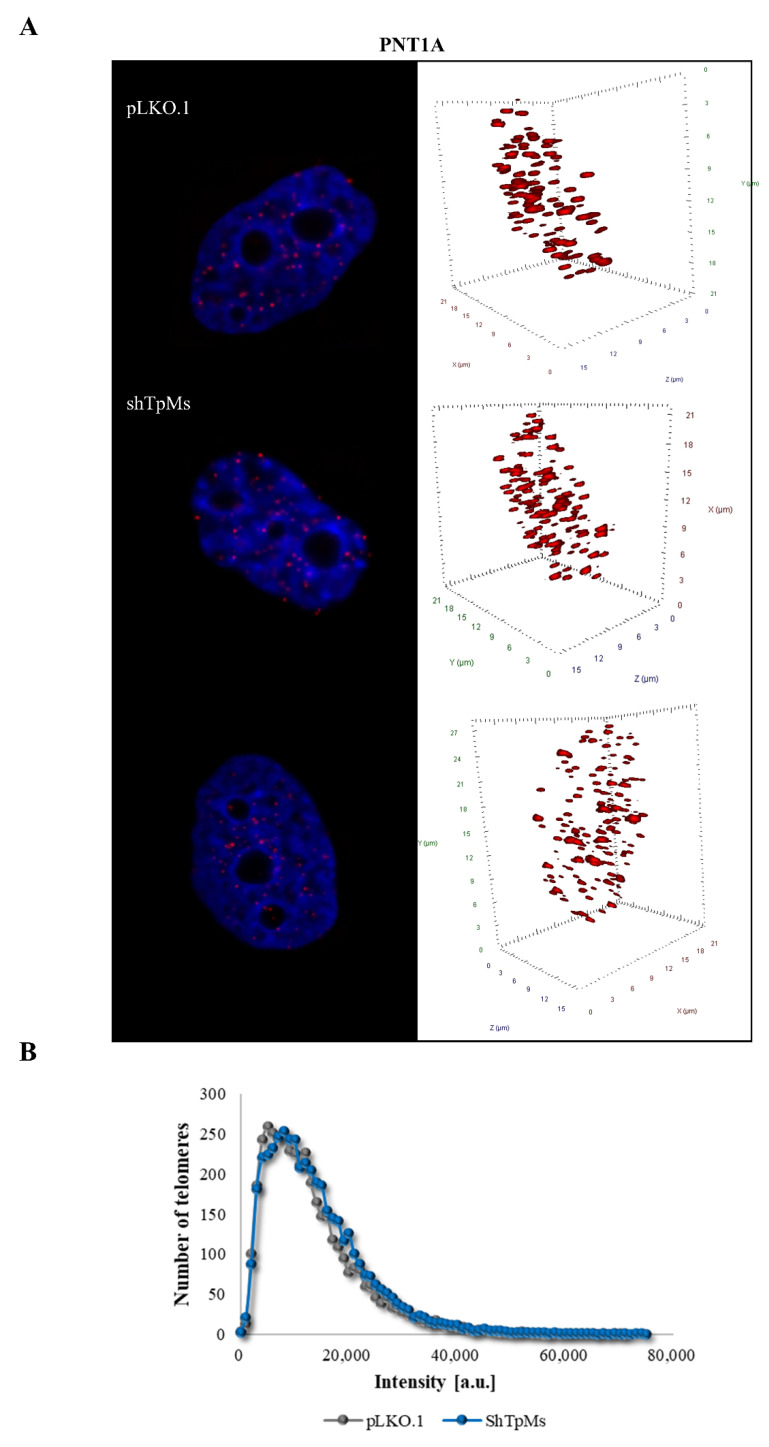
Number of telomere signals and aggregates in TpMs-depleted PNT1A cells. (**A**) Representative nuclei, counterstained with DAPI (4′,6-diamidino-2-phenylindole) (blue), where Cy-3 labelled telomeres appear as red dots. (**B**) A telomere intensity histogram showing distribution of signal intensities in PNT1A cells depleted of TpMs with respect to control. Loss of TpMs in PNT1A cells did not show significant differences in the number of telomere signals and aggregates compared with the control cells (pLKO.1). [a.u.] = arbitrary units. Abscissa = intensity [a.u]. ordinate = number of telomere signals.

Contrary to what we observed in the TpMs-depleted HCT116 cells, in the TpMs-depleted PNT1A cells, the number of telomere signals (Figure 3A,E and Figure 4), the number of the aggregates (Tas) (Figure 3B,E), the telomere intensity (Figure 3C,E), and the telomere distribution indicated by the a/c ratio (Figure 3D,E) did not show significant differences compared with the control cells (pLKO.1) (*p* = 0.0129). These findings are in accordance with a previous study [34], in which the telomere dysfunctions associated with chromosome instability were blocked in cells immortalized with SV40.

### 3.2. TpMs-Depleted HCT116 Cells Display Differences in Nuclear DNA Organization

The organization of the nuclear architecture is important to maintain the stability of the cellular genome. Any change that alters nuclear organization potentially induces variations in its organization that precede the onset of genomic instability [29].

Cancer is a disease of DNA organization [38]. It is known that the nuclear architecture differs between normal and cancer cells. Observations by Mai et al. previously demonstrated differences between normal and cancer cell nuclei in both the DNA structure and DNA-free space together with both nuclear and nucleolar remodeling [32]. The DNA-poor space refers to the interchromatin space visualized by super resolution imaging. This is distinct from the nucleoli.

We checked whether TpMs loss induces changes in the genome organization. To analyze the DNA distribution within the nucleus and to determine whether there are differences between TpMs-depleted cells and control cells (pLKO.1), we imaged HCT116 cells (stained by 4′,6-diamidino-2-phenylindole (DAPI)) using 3D Structured Illumination Microscopy (SIM), a super-resolution imaging modality that offers a higher image resolution than conventional epifluorescence wide-field microscopy [39,40,41]. This technology captures the size and intensity of fluorescent DNA signals in three dimensions, revealing the size distribution of DNA structures and DNA-poor spaces in super-resolution imaging. We used granulometry to evaluate the size distribution of the DNA structure and the DNA-free space, by light and dark granulometry, respectively [33].

Granulometry measures the diameters of granule-like regions in DNA and DNA-free spaces, resulting in cumulative size distributions of DNA structure and DNA-free space [33].

**Figure 5:**
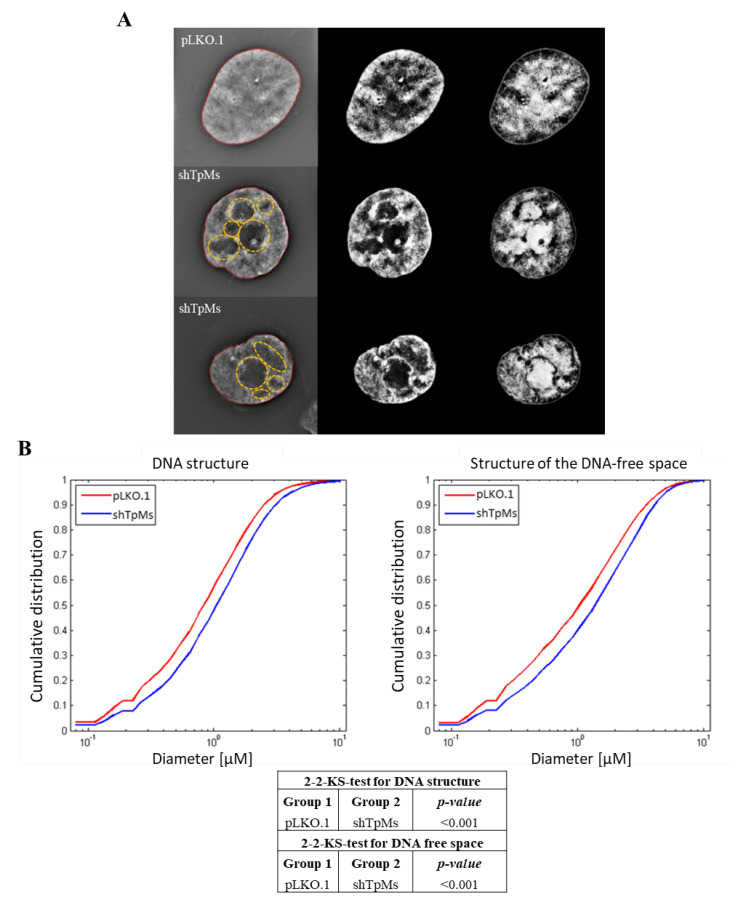
TpMs−depleted cells present different DNA structure and content in the DNA-free space. (**A**) Representative images from DAPI-stained nuclei of TpMs-depleted HCT116 cells (middle and bottom rows in the panel) and pLKO.1 control cells (top row). The scale bars are 5 µm in each image. Left panels: reconstructed 3D−SIM images; middle panels: light granulometry images; right panels: dark granulometry images. Images acquired with 63× Oil immersion objective. (**B**) Comparisons of cumulative size distribution of the DNA structure and DNA-free space between the two groups (pLKO.1 and shTpMs). All these size distributions are measured with a granulometry 2−2−KS-test (Two-Sample Kolmogorov-Smirnov Test)*, p*-value < 0.001.

After acquisition of HCT116 cells and image reconstruction, we determined the intra-nuclear DNA structure. We analyzed a total of ninety interphase TpMs-depleted nuclei at three different passages in comparison with control nuclei. We found that TpMs-depleted cells present a larger amount of submicron DNA structure (*p* = 3.7 × 10^−6^) and display larger open areas/holes (larger DNA-free space) (*p* = 2.13 × 10^−6^) compared with control cells (pLKO.1) (Figure 5A,B). The results indicate different intra-nuclear organization between TpMs-depleted (shTpMs) and control cells (pLKO.1). This type of event is generally believed to be a marker of cellular transformation [33].

## 4. Discussion

In vitro and in vivo data demonstrate that TpMs protein plays diverse functions in eukaryotic cells that depend on the intracellular localization of the protein, ranging from microtubule-anchor activity at the centrosome [3], negative regulation on primary cilia assembly in the G1 phase [5] to tumor suppression function [2]. TpMs negatively affects cell growth, while it is markedly downregulated in advanced stages of mammary and bladder carcinomas [2].

There is a general consensus that TpMs shows the hallmarks of a classical tumor suppressor [42]. Many efforts are aiming to investigate the basic roles of TpMs in solid tumor onset and progression.

Holdgaard et al. [43] recently have identified doryphagy as an important centrosome-regulating pathway and bring mechanistic insights to the link between autophagy dysfunction and chromosomal instability.

Recently, we demonstrated that TCHP (TpMs) regulates endothelial cell function via an autophagy-mediated mechanism [8]. Furthermore, our recent findings highlighted a novel role for TpMs, both as a mitotic checkpoint regulator and a guardian of chromosomal stability [9]. TpMs interacts with Mad2, and TpMs depletion results in decreased levels of Mad2 and Cyclin B1 proteins, followed by chromosome mis-segregation, DNA damage and chromosomal instability. Similarly to our results, previous studies have reported various mitotic defects, such as failure in chromosome segregation, premature anaphase onset, and cytokinesis alterations in Mad2 or BubR1-depleted cells [44,45].

Here, we show that silencing of TpMs expression in the HCT116 cell line results in telomere dysfunction. Our telomere function studies allow the linking of decreased telomere intensity with proliferation, which is indicative of mitotic slippage, and telomeric aggregates maintenance, which is indicative of genomic instability [9,21,22]. It is noteworthy that TpMs-depleted PNT1A cells, a normal prostatic epithelial cell line immortalized with SV40, do not display telomere dysfunction, which is in accordance with a previous study [34]. Although we examined telomere dysfunction, we have not studied the role of shelterin proteins under conditions of TpMs depletion. Shelterin proteins cap the telomeric ends of chromosomes and protect them from fusion and illegitimate recombination events [46]. Future studies will clarify how TpMs affects shelterin proteins and telomere (un)capping. Subsequently, we utilized 3D Structured Illumination Microscopy (SIM) to evaluate the genome organization in TpMs absence. We found a change in the DNA structure, with an increase in DNA-poor interchromatin space in TpMs-depleted HCT116 cells compared with controls (Figure 5), thus indicating a different intra-nuclear organization between TpMs-depleted and control cells.

This event is unanimously believed to be a marker of cellular transformation [33]. The aberrations we observed might result from defective chromosomal segregation [47] that, in turn, is consistent with a defective mitotic checkpoint [9]. Generally, both numerical chromosomal aberrations (aneuploidy) and/or structural chromosomal aberrations (translocations, deletions, breaks, triradial complexes) that are detected in human tumors are connected to the tumorigenesis process. Chromosomal instability (CIN) originates from aberrant mitotic divisions and is a typical characteristic of cancer onset and development. Impairment of the mitotic checkpoint response is included among the diverse mechanisms that generate aberrant mitotic divisions [9]. Several types of human tumor cells have diminished mitotic checkpoint responses that are often associated, together with aneuploidy, with changes in the levels of mitotic checkpoint proteins [48]. Mouse models in which mitotic checkpoint signaling is decreased show increased spontaneous or carcinogen-induced tumor formation [48]. Similarly, Baker et al. have demonstrated that elevated chromosomal instability may drive tumor onset and progression by promoting the loss of additional tumor suppressor genes and possibly also by facilitating the gain of important oncogenes [49]. In conclusion, in our previous study [9], we identified for the first time that TpMs depletion causes an impaired cell spindle assembly checkpoint, and consequent events such as chromosome misalignment and mis-segregation together with increased chromosomal instability. We demonstrated that TpMs play a critical role in guarding the fidelity of mitosis by enabling the optimal activation of the spindle checkpoint. Furthermore, here we report that TpMs-depleted HCT116 cells show telomere dysfunctions associated with chromosome instability, and display differences in nuclear DNA organization. We have started from findings that TpMs is implicated in the malignant progression of several human tumors [2] to hypothesize that the observed TpMs role in the maintenance of chromosome stability [9] may contribute to its tumor suppressor function [2,21,22].

## Data Availability

Data regarding this study are available on request to the corresponding authors.

## Supplementary Materials

The following supporting information can be downloaded at: https://www.mdpi.com/article/10.3390/biomedicines10071602/s1, Figure S1: HCT116 and PNT1A cells stably transfected with lentiviral particles harboring TpMs shRNA (Sh-TpMs) and control shRNA (pLKO.1), Figure S2: Original blots and densitometry quantification. HCT116 and PNT1A cells stably transfected with lentiviral particles harboring TpMs shRNA (Sh-TpMs) and control shRNA (pLKO.1). Video S1: The full z-stacks of telomere signals for TpMs-depleted (shTpMs) and control (pLKO.1) cells.
