# Supplementary material for: Telomere Dysfunction Is Associated with Altered DNA Organization in Trichoplein/Tchp/Mitostatin (TpMs) Depleted Cells
Source: Biomedicines. 2022 Jul 5;10(7):1602. doi: 10.3390/biomedicines10071602 (PMC9312488; doi:10.3390/biomedicines10071602)
Supplement: Supplementary file 1 [file biomedicines-10-01602-s001.zip › biomedicines-1787732-Supplementary/biomedicines-1787732-Supplementary .pdf]

Telomere Dysfunction Is Associated with Altered DNA Organization in Trichoplein/Tchp/Mitostatin (TpMs) Depleted Cells

Angela Lauriola [1], Pierpaola Davalli [2], Gaetano Marverti [2], Andrea Caporali [3], Sabine Mai [4,*] and Domenico D'Arca [2,*]

[1] Department of Biotechnology, University of Verona, 37134 Verona, Italy; angela.lauriola@univr.it
[2] Department of Biomedical, Metabolic and Neural Sciences, University of Modena and Reggio Emilia, Via G. Campi 287, 41125 Modena, Italy; pierpaola.davalli@unimore.it (P.D.); gaetano.marverti@unimore.it (G.M.)
[3] The Queen's Medical Research institute, BHF Centre for Cardiovascular Science, University of Edinburgh, EH104AH Edinburgh, UK; acaporal@exseed.ed.ac.uk
[4] CancerCare Manitoba Research Institute, University of Manitoba, CancerCare Manitoba, Winnipeg, MB R3E 0V9, Canada
* Correspondence: sabine.mai@umanitoba.ca (S.M.); domenico.darca@unimore.it (D.D.); Tel.: +1-204-272-3174 (S.M.); +39-059-205-5610 (D.D)

Supplementary Material

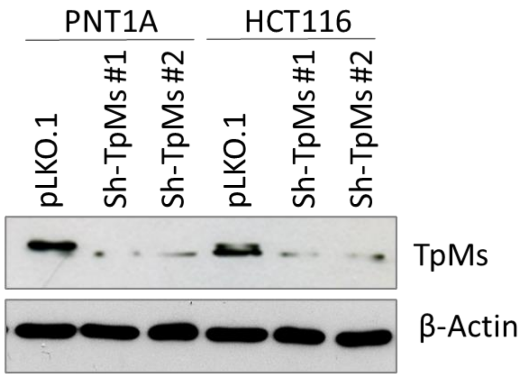

Figure S1. HCT116 and PNT1A cells stably transfected with lentiviral particles harboring TpMs shRNA (Sh-TpMs) and control shRNA (pLKO.1).

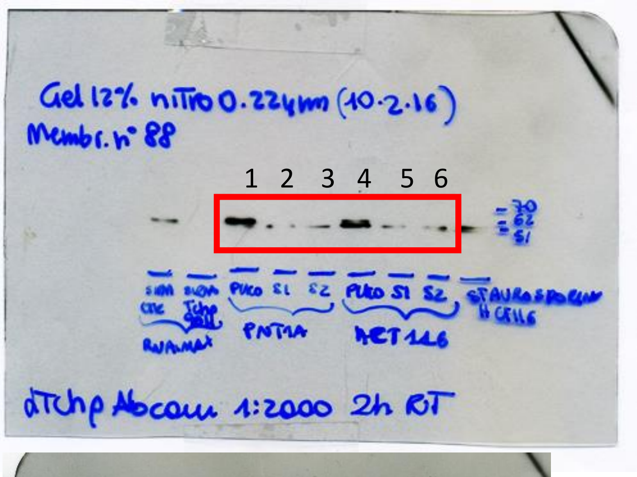

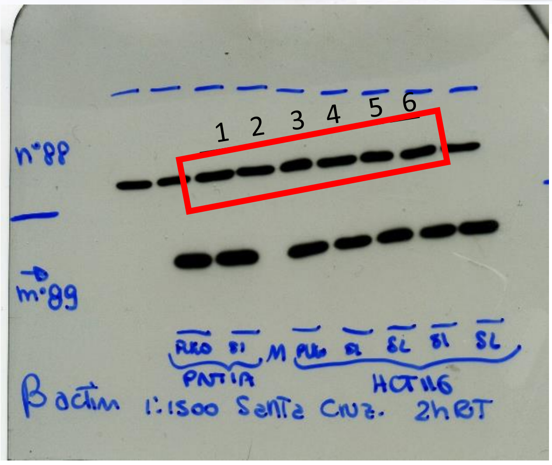

Samples
1. PNT1A pLKO.1
2. PNT1A Sh-TpMs #1
3. PNT1A Sh-TpMs #2
4. HCT116 pLKO.1
5. HCT116 Sh-TpMs #1
6. HCT116 Sh-TpMs #2

Densitometry	
**Sample**	**TpMs/Actin**
PNT1A pLKO.1	0,81
PNT1A Sh-TpMs #1	0,06
PNT1A Sh-TpMs #2	0,08
HCT116 pLKO.1	0,73
HCT116 Sh-TpMs #1	0,06
HCT116 Sh-TpMs #2	0,09

Figure S2. Original blots and densitometry quantification. HCT116 and PNT1A cells stably transfected with lentiviral particles harboring TpMs shRNA (Sh-TpMs) and control shRNA (pLKO.1).
